# Enhanced Antibacterial Activity of Dermaseptin through Its Immobilization on Alginate Nanoparticles—Effects of Menthol and Lactic Acid on Its Potentialization

**DOI:** 10.3390/antibiotics11060787

**Published:** 2022-06-09

**Authors:** Noura Hazime, Yanath Belguesmia, Alexandre Barras, Mohamed Amiche, Rabah Boukherroub, Djamel Drider

**Affiliations:** 1 Univ. Lille, CNRS, Centrale Lille, Univ. Polytechnique Hauts-de-France, UMR 8520, IEMN, F-59000 Lille, France; noura.hazime@hotmail.fr (N.H.); alexandre.barras@univ-lille.fr (A.B.); rabah.boukherroub@univ-lille.fr (R.B.); 2 UMR Transfrontalière BioEcoAgro1158, Univ. Lille, INRAE, Univ. Liège, UPJV, JUNIA, Univ. Artois, Univ. Littoral Côte D’Opale, ICV—Institut Charles Viollette, F-59000 Lille, France; yanath.belguesmia@univ-lille.fr; 3 Laboratoire de Biogenèse des Signaux Peptidiques (BioSiPe), Institut de Biologie Paris-Seine, Sorbonne Université—CNRS, F-75252 Paris, France; mohamed.amiche@upmc.fr

**Keywords:** dermaseptin, DRS-B2, alginate nanoparticles, menthol, lactic acid, synergy, potentialization, cytotoxicity

## Abstract

Dermaseptin B2 (DRS-B2) is an antimicrobial peptide secreted by *Phyllomedusa bicolor*, which is an Amazonian tree frog. Here, we show that the adsorption of DRS-B2 on alginate nanoparticles (Alg NPs) results in a formulation (Alg NPs + DRS-B2) with a remarkable antibacterial activity against *Escherichia coli* ATCC 8739 and *E. coli* 184 strains, which are sensitive and resistant, respectively, to colistin. The antibacterial activity, obtained with this new formulation, is higher than that obtained with DRS-B2 alone. Of note, the addition of lactic acid or menthol to this new formulation augments its antibacterial activity against the aforementioned Gram-negative bacilli. The safety of DRS-B2, and also that of the new formulation supplemented or not with a small molecule such as lactic acid or menthol has been proven on the human erythrocytes and the eukaryotic cell line types HT29 (human) and IPEC-1 (animal). Similarly, their stability was determined under the conditions mimicking the gastrointestinal tract with different conditions: pH, temperature, and the presence of digestive enzymes. Based on all the obtained data, we assume that these new formulations are promising and could be suggested, after *in vivo* approval and completing regulation aspects, as alternatives to antibiotics to fight infections caused by Gram-negative bacilli such as *E. coli*.

## 1. Introduction

Dermaseptins (DRS) are a large family of polycationic antimicrobial peptides (AMPs) isolated from the skin secretions of tree frogs belonging to *Phyllomedusa* genus [1]. These α-helical structured peptides, which contain between 24 and 34 amino acids, are rich in irregularly spaced lysine (Lys) residues, and usually contain a tryptophan (Trp) residue in position 3 of their sequence. Besides these features, DRS peptides contain a central AGKAAL motif sequence [2]. These peptides are active against a panel of Gram-positive and Gram-negative bacteria, as well as against yeasts, protozoa, and fungi and act generally by disruption of the lipid bilayer of target cells [3]. Besides their large spectrum of activity, DRS are not hemolytic [3]. DRS-S1 is considered as the first member of the DRS family, and this peptide was isolated from dried skin extracts of *Phyllomedusa sauvagei* [4], whereas the second member, which is DRS-B2, was isolated from the exudate of *Phyllomedusa bicolor* [5,6,7]. DRS-B2 is also known as an adenoregulin because of its ability to regulate adenosine A1 receptor agonist binding [8]. DRS-B2 has the highest activity in the DRS family and is therefore the most studied peptide. DRS-B2 is an amphipathic (+3) cationic polypeptide containing 33 amino acids, with a molecular weight of 3180 Da, a tryptophan residue at position 3, and six lysine residues. DRS-B2 is active against Gram-positive and Gram-negative bacteria, yeasts, protozoa and filamentous fungi [6,9,10]. A previous study showed the activity of DRS-B2 against *E. coli* strains including *E. coli* B [11] and *E. coli* K12 [5,9]. Other studies aimed at improving this activity have focused on the relationship between the structure and function of DRS-B2 [3,9]. Thus, the N-terminal region proved to be essential for this activity, unlike the C-terminal regions DRS-B2-(1–23) and DRS B2-(1–11), and its alteration only causes a reduction but not the loss of activity. Nonetheless, the full sequence of this peptide is required to obtain a complete permeabilization of the membrane and therefore a bacterial lysis. Moreover, few studies have shown anti-malaria activities of both DRS-S3 and S4 within the erythrocyte, without causing any alteration of host functions [12,13,14]. Related to the antimicrobial activities of DRS, several studies have shown their in vitro antifungal activities [15], particularly for DRS-S3, which targets the phenomenon of apoptosis [16]. DRS are active against a wide variety of pathogens and tumor cells, therefore supporting their potential application as new drugs to treat bacterial infections or to strengthen conventional chemotherapies in cancer treatments [2]. This family of AMP has also been suggested as a new antidiabetic drug for treatment of type 2 diabetes disease [17]. Immunomodulatory effect was also established for dermaseptin-01, which appeared to increase the phagocytic capacity and production of hydrogen peroxide by the macrophages in a BALB/c mice model [18].

The present report emphasizes the antibacterial activity of DRS-B2 against Gram-negative bacilli. We show that the adsorption of DRS-B2 on alginate nanoparticles (Alg NPs) leads to a new and potent antibacterial formulation designed as Alg NPs + DRS-B2. Alginate is a suitable polymer able to drive drugs to the different sites of infection. This safe, biodegradable, and economically-affordable polymer has been found to be a mucoadhesive and non-immunogenic substance [19,20,21,22,23]. Of note, the optimal loading of proteinaceous AMPs such as bacteriocins on Alg NPs has resulted in a formulation with improved antibacterial activity compared to the use of bacteriocin alone [24,25]. Here, we show that the optimal loading of DRS-B2, which is a member of the dermaseptin family, on Alg NPs also results in a more active formulation compared to DRS-B2 alone when assessed against *Escherichia coli,* including strains displaying a resistant phenotype to colistin. Likewise, the incorporation of small molecules such as menthol or lactic acid to the previously developed formulation (Alg NPs + DRS-B2) allows to further improve the antibacterial activity against Gram-negative bacilli. Consequently, we demonstrate that alginate has a potentiating role on the antibacterial activity of DRS-B2 and menthol or lactic acid, which potentiating effects have already been established [26,27] and can be added to the design of these new formulations.

In this study, we thus highlight that the antibacterial activity of the DRS-B2 peptide could be enhanced by its adsorption on Alg NPs and the addition of small molecules such as menthol or lactic acid. These formulations, whose safety and stability have also been studied, may constitute good candidates for therapeutic applications in order to replace the use of fading antibiotics in certain practices.

## 2. Material and Methods

### 2.1. Bacterial Strains

The *Escherichia coli (E. coli)* strains used in this study included *E. coli* ATCC 8739 and *E. coli* 184, which is resistant to colistin. *E. coli* 184 was obtained from the French Résapath network for the surveillance of antimicrobial resistance in the pathogenic bacteria of animal origin (https//www.resapath.anses.fr/, accessed on 18 May 2022) and was shown to carry the *mcr-1* gene responsible for resistance to colistin. *E. coli* strains were grown at 37 °C in Brain Heart Infusion (BHI) medium (Sigma-Aldrich, St. Louis, MA, USA).

### 2.2. Elaboration of Alginate Nanoparticles (Alg NPs)

Alg NPs were prepared by a top-down process using a ball milling planetary mixer PM100 (Retsch GmbH, Haan, Germany), according to the protocol described by Zgheib et al. [24].

### 2.3. Preparation of Alg NPs Loaded with Dermaseptin (DRS-B2) and Small Molecules (Menthol or Lactic Acid)

The synthesis of DRS-B2, whose amino-acid sequence is GLWSKIKEVGKEAAKAAAKAAGKAALGAVSEAV-CONH_2_, was performed with a solid-phase 9-fluorenylmethoxycarbonyl (Fmoc) chemistry procedures using an automated microwave-assisted peptide synthesizer liberty 1 (CEM Corporation, Charlotte, NC, USA) [28]. Formulations composed of Alg NPs + DRS-B2 or Alg NPs + DRS-B2 with any small molecule were prepared at different concentrations (Table 1). The concentration of Alg NPs has been set to 500 µg/mL in order to allow a good dispersion and avoid any aggregation or sedimentation that could interfere with the adsorption [24]. Of note, the pH of all Alg NPs formulations was adjusted to pH 7. The size and charge of Alg NPs + DRS-B2 were determined by the dynamic light scattering (DLS) method and the zeta potential, as previously described [25].

### 2.4. Preparations of Different DRS-B2 Formulations for HPLC Analyses

DRS-B2 solutions of different concentrations were used to establish a standard curve, allowing determination of the amount of DRS-B2 adsorbed on Alg NPs by the high-performance liquid chromatography (HPLC) method. HPLC measurements were performed for each solution, with and without Alg NPs. DRS-B2 solutions at the following concentrations: 3.5, 30, 46, 57, 90, 129, 157, 190, 199, 225, and 250 µg/mL, were prepared by dilution in distilled water and were then homogenized by sonication for 45 min at 25 °C.

For each combination with Alg NPs, two series of formulations were prepared with different ratios of DRS-B2/Alg NPs. One set was stored at 4 °C and the other one was dialyzed against a 50 kDa membrane for 24 h at room temperature to separate free peptides from adsorbed ones. At the end of the dialysis, solutions from each batch were recovered for HPLC analyses. Thus, 40 µL of each solution were injected into the HPLC column (5 µm QS Uptisphere^®^ 300 Å, 250 mm × 4.6 mm C4 column, Interchim, Montluçon, France), using as a mobile phase a solution composed of eluent A (0.1% trifluoroacetic acid in deionized water) and eluent B (0.1% trifluoroacetic acid in acetonitrile 100%). The flow rate used was 1 mL/min. The temperature of the column was set to 40 °C, and the elution was conducted with a linear gradient ranging from 0 to 80% for 30 min. The sample was filtered through a 0.45 µm cellulose membrane (VWR, Radnor, PA, USA) and then loaded on the HPLC column. The detection was realized at 215 and 254 nm. The calibration curve was obtained by a linear regression of known concentrations of DRS-B2 (µg/mL) against the measured peak area obtained after the HPLC analyses.

### 2.5. Determination of Minimal Inhibitory Concentrations

The minimum inhibitory concentration (MIC) of the aforementioned formulations was determined in triplicate in 96-well microtiter plates using the broth microdilution method [29]. Fresh colonies of *E. coli* were sub-cultured in Mueller–Hinton broth (Sigma-Aldrich) and were grown at 37 °C overnight on a rotary shaker at 160 rpm. MIC values were determined in 96-well round-bottom plates using a volume of 250 μL of Mueller–Hinton broth containing the formulations at different concentrations, inoculated with 2.5 µL of the *E. coli* strain’s pre-culture. The plates were incubated for 24 h at 37 °C, and inhibition of growth was measured at 600 nm with a spectrophotometer ELx808^tm^ (BioTek, Winoosky, VT, USA). The MIC was determined as the lowest concentration of DRS-B2 peptide allowing the absence of a visible growth, compared to non-inoculated medium used as a control.

### 2.6. Hemolysis

The hemolytic activities of Alg NPs (0.002 mg/mL to 2.000 mg/mL) or DRS-B2 (0.026 mg/mL to 0.208 mg/mL) or Alg NPs (0.500 mg/mL) with DRS-B2 (0.002 mg/mL to 0.208 mg/mL) were determined on fresh human erythrocytes obtained from a healthy donor, as previously described [30]. Cells treated with 0.2% Triton X100 were used as a positive control (CTL+) and correspond to the total (100%) of hemolytic activity.

### 2.7. Cytotoxicity Assay

The cytotoxicity assay was performed as previously reported [31]. Briefly, human colon carcinoma (HT29) and porcine intestinal (IPEC-1) cells (Sigma-Aldrich) were cultivated in Dulbecco’s Modified Eagle Medium (DMEM, Gibco, Thermo Fisher, Waltham, MA, USA) in 96-well-tissue culture plates for 48–72 h at 37 °C, in atmosphere containing 5% CO_2_, until the formation of a continuous confluent cell culture on the bottom of each well. DRS-B2 and formulations corresponding to DRS-B2 loaded on Alg NPs, combined or not with small molecules (menthol or lactic acid), were tested at their MIC values. The required concentrations were prepared in DMEM without antibiotics and serum and were added to the HT29/IPEC-1 cells in the wells, after washing with the same medium. The treated cells were then incubated for 24 h at 37 °C, in atmosphere containing 5% CO_2_. To assess the cell viability of the treated HT29/IPEC-1 cells, CCK8 assay (Dojindo Molecular Technologies, Tokyo, Japan) was realized according to the supplier recommendations. An amount of 150 µL of DMEM containing 7.5 µL of CCK-8 reagents were added to each well, and cells were incubated for 2 h. Plates were read at 450 nm in a microplate reader spectrophotometer (Xenius, Safas, Monaco). Results were expressed in % of basal growth observed with non-treated cells.

### 2.8. In Vitro Stability of the Alg NPs-Based Formulations in Conditions Mimicking the Gastrointestinal Tract

The ability of these new formulations to resist the conditions of the gastrointestinal tract (GIT) environment was determined as previously described [32]. These GIT-simulated conditions include a gastric compartment mimicking solution, which contains 15 U/mL of pepsin (Sigma-Aldrich) dissolved in 20 mM PBS adjusted at pH 3 with 0.5 M HCl, and incubated at 37 °C with continuous agitation (160 rpm) for 30 min, followed by simulating the duodenal compartment solution by adding 40 U/µL of trypsin and 5 U/mL of chymotrypsin (Sigma-Aldrich) (pH 6) in 20 mM PBS and an additional 2 h of incubation under similar conditions. All these new formulations, including DRS-B2 loaded on Alg NPs associated or not with menthol (10 µg/mL) or lactic acid (1 µg/mL), were tested for their stabilities under GIT-simulated conditions. After each step, a sufficient volume was withdrawn to assess the remaining antibacterial activity against *E. coli* 184 strain, as described above.

### 2.9. Statistical Analysis

The data are expressed as means ± SD (standard-deviation), calculated from three independent experiments (*n* = 3). The data were subjected to one-way analysis of variance (ANOVA). Tukey’s test was employed to determine the significant differences between the variables at *p* < 0.05.

## 3. Results

### 3.1. Quantification of DRS-B2 Adsorbed on Alg NPs by HPLC

The maximum quantity of DRS-B2 adsorbed on the surface of the Alg NPs was determined by HPLC analyses. The results obtained for DRS-B2 are presented in Supplementary Figure S1 and Table 2.

The differences in the concentration of DRS-B2 observed before and after dialysis could be ascribed to the exclusion of this peptide by the membrane. Related to that, the dialysis membrane used has a cut-off of 50 kDa, excluding, therefore, the passage of Alg NPs, whose sizes range from 99 to 200 nm (data not shown), but allowing the diffusion of non-adsorbed DRS-B2 peptide.

For concentrations from 3.5 to 190 µg/mL, areas of the peaks before and after dialysis have increased with a difference of ~16.8%, indicating that almost all DRS-B2 was adsorbed on the surface of Alg NPs. According to the data presented in Figure S1 and those obtained from the calibration curve (Y = 19931X), the upmost concentration of DRS-B2 adsorbed on the surface of the Alg NPs (500 µg/mL) was 200 µg/mL (Table 2).

To sum up, the highest concentration of DRS-B2 adsorbed on the surface of the Alg NPs (500 μg/mL) is 0.4 µg/µg (Table 2). DRS-B2 adsorption on Alg NPs was further confirmed by dynamic light scattering (DLS) and zeta potential measurements. In fact, the median diameter size of the Alg NPs increased from 111 to 128 nm after DRS-B2 adsorption. This result was in addition corroborated by the change of the negative surface charge of bare Alg NPs (−22 mV, pH = 6.3) to a positive value (+0.9 mV, pH = 6.3) after DRS-B2 adsorption, indicating the neutralization of the negative charges on the Alg NPs upon adsorption of the positively-charged DRS-B2 peptide (ζ = +23 mV at pH = 6.3) (Table S1).

### 3.2. Determination of the Antibacterial Activity of Alg NPs + DRS-B2 + Small Molecules Formulations

The antibacterial activity of DRS-B2 against *E. coli* 184 and ATCC8739 was first established by using the well-known agar diffusion method (data not shown). The MIC values obtained with DRS-B2 alone were 7.5 µg/mL (*E. coli* 184) and 3.75 µg/mL (*E. coli* ATCC 8739) (Table 3).

The adsorption of DRS-B2 on Alg NPs results Alg NPs + DRS-B2. This new formulation has a remarkable antibacterial activity unlike DRS-B2 alone. As indicated in Table 3, the MIC values of DRS-B2 (40 µg/mL) decreased from 7.5 to 2.5 µg/mL (*E. coli* 184) and from 3.75 to 1.25 µg/mL (*E. coli* ATCC8739).

The addition of menthol at a non-cytotoxic concentration (10 µg/mL) to the formulation Alg NPs + DRS-B2 decreased the MIC values at least two-fold, in comparison to those recorded for the formulation Alg NPs + DRS-B2 and, noteworthy, by six-fold in comparison to DRS-B2 alone. Clearly, Alg NPs or Alg NPs + menthol potentiates the antibacterial activity of DRS-B2, and the MIC values decreased from 2.5 to 1.25 µg/mL (*E. coli* 184), and from 1.25 to 0.62 µg/mL (*E. coli* ATCC8739), as indicated on Table 4.

The addition of lactic acid, at 1 µg/mL, to the formulation Alg NPs + DRS-B2 also enabled the decrease of MIC values by two-fold against both *E. coli* strains (Table 5). Similarly, the addition of lactic acid at this non-inhibitory concentration also has a potentiating effect on the DRS-B2 activity.

### 3.3. Hemolytic Activity of DRS-B2, Alg NPs, and Alg NPs + DRS-B2

**Figure 1:**
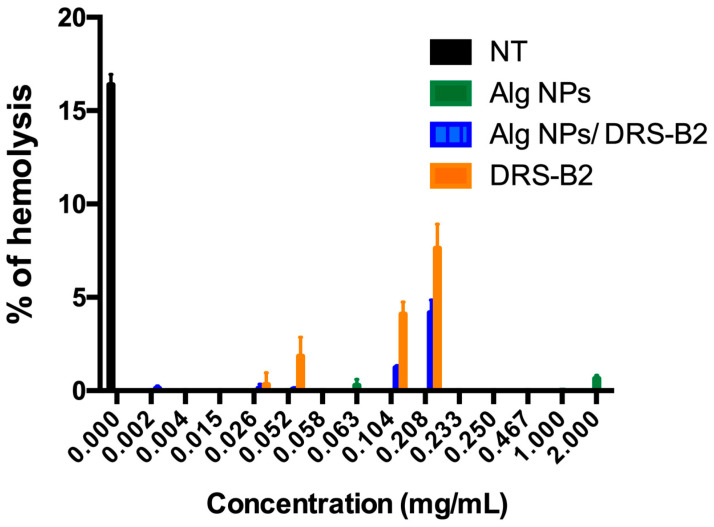
Hemolytic activity of Alg NPs, DRS-B2 alone, and Alg NPs + DRS-B2 on human erythrocytes. Human erythrocytes were cultured without treatment (NT) or upon treatments with different concentrations of Alg NPs, DRS-B2, or Alg NPs (0.500 mg/mL) + DRS-B2 (0.002–0.208 mg/mL) for 1 h at 37 °C.

The safety of the different formulations was ascertained on the human erythrocytes. Indeed, no hemolytic activity was detected upon incubation of the human erythrocytes with different concentrations of Alg NPs containing DRS-B2 or not (Figure 1).

Remarkably, Alg NPs at 2000 µg/mL had no hemolytic activity on the human erythrocytes, and the percentage of hemolysis obtained was below 0.5% (≤0.5%). Meanwhile, DRS-B2 (0.208 mg/mL) displayed a hemolysis calculated to be below 10% (≤10%), compared to the percentage of the non-treated (NT) samples. Remarkably, the combination of DRS-B2 (0.208 mg/mL) and Alg NPs (0.500 mg/mL) enabled the decrease of this hemolysis below 5% (Figure 1).

According to the data displayed in Figure 1, Alg NPs did not exhibit any hemolytic activity on human red blood cells, and their combination with DRS-B2 permitted it to lower down the hemolytic activity of DRS-B2 from 8% to 4%.

### 3.4. Assessment of Cytotoxicity on IPEC-1 and HT29 Cell Lines

**Figure 2:**
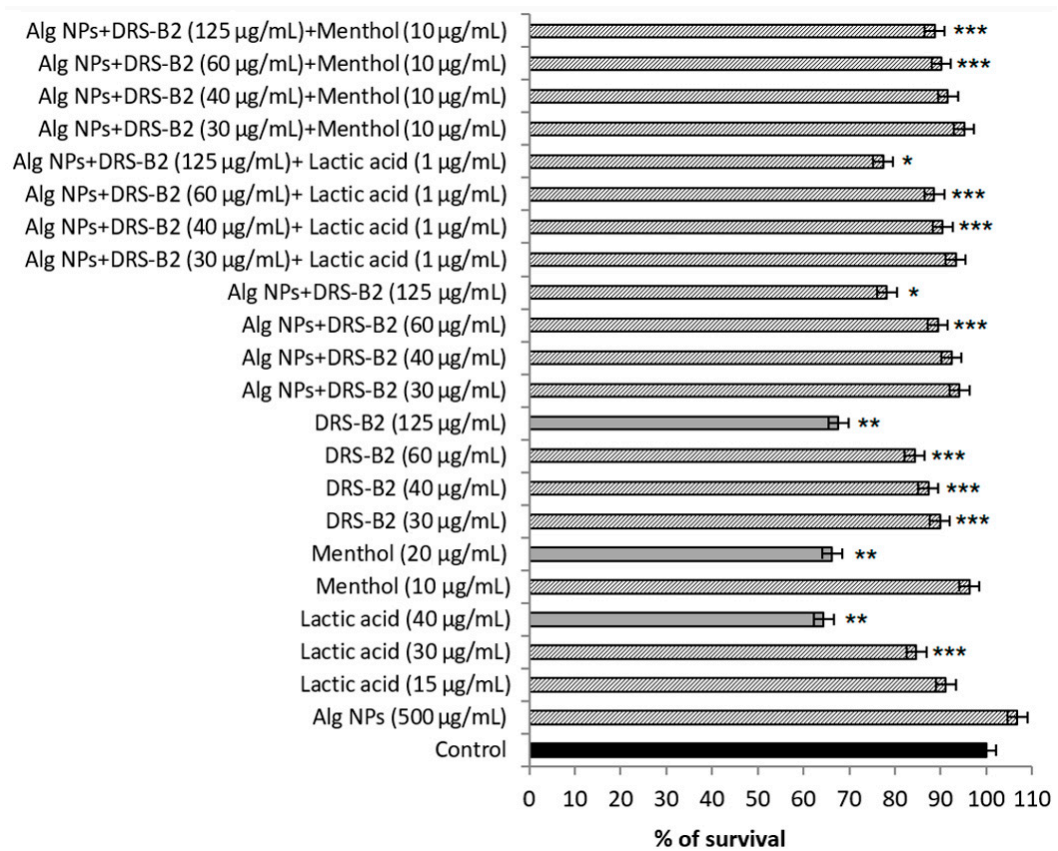
Cytotoxicity of DRS-B2 and the different formulations on porcine IPEC-1 cell line. Means with (*), (**), or (***) are significantly different from control and between them (*p* < 0.05).

**Figure 3:**
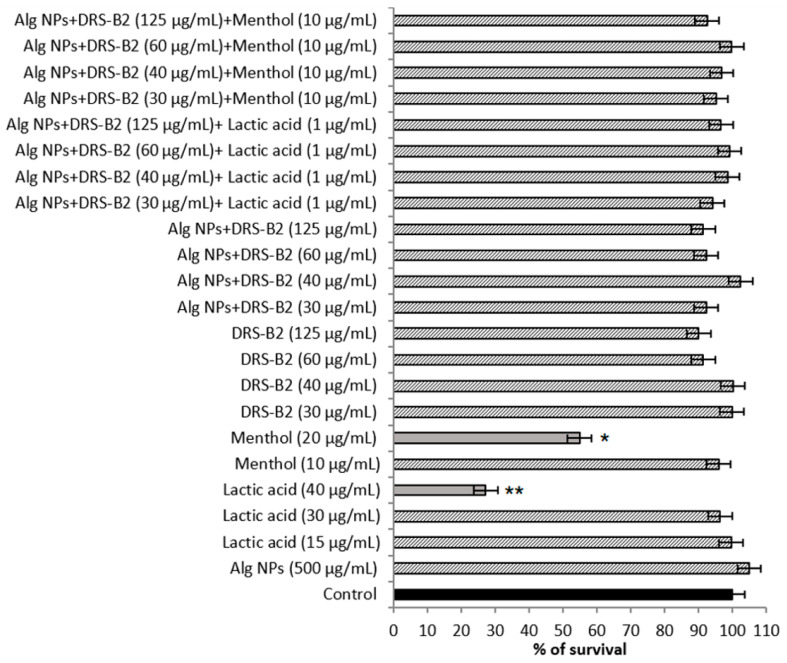
Cytotoxicity of DRS-B2 and different formulations on human HT29 cell line. Means with (*) or (**) are significantly different from control and between them (*p* < 0.05).

The cytotoxicity was determined on two eukaryotic cell lines, which were IPEC-1, a porcine cell line, and HT29, a human cell line (Figure 2 and Figure 3).

As depicted in Figure 2, the percentage of survival of IPEC-1 cell line obtained in the presence of the DRS-B2 peptide at 30 to 125 µg/mL was between 87% and 68%, respectively. Small molecules such as menthol and lactic acid used at 20 and 40 µg/mL showed comparable data, with survival rates of 66% and 64%, respectively. Interestingly, the survival rates are augmented significantly when DRS-B2 was adsorbed on Alg NPs (Alg NPs + DRS-B2), reaching 90% and 78%, respectively. It should be noted that Alg NPs (500 µg/mL) is safe and enabled 100% survival. As seen in Figure 3, similar data were obtained on the HT29 cell line.

DRS-B2 is not toxic on the HT29 cell line, even at high concentrations. The cell viability recorded under these conditions was between 90% and 100% (Figure 3). Nonetheless, menthol and lactic acid at high concentration of 20 and 40 µg/mL, respectively, could alter the cell viability because the survival rates obtained were under 50%. These concentrations are, however, much higher than those used in the development of new and appropriate formulations (10 µg/mL for menthol and 1 µg/mL for lactic acid).

### 3.5. Stability of DRS-B2 + Alginate Nanoparticles after Enzymatic Treatment

The impact of digestive enzymes on the activity of Alg NPs + DRS-B2 with lactic acid (1 µg/mL) or menthol (10 µg/mL) was tested against *E. coli* 184 during two incubation times and at two pH values (pH 3 and pH 6). To this end, we used pepsin, a digestive enzyme from the stomach, and active at pH 3, trypsin and chymotrypsin, which are synthesized in the pancreas and secreted in the small intestine, active between pH 6 and pH 8.

Of note, DRS-B2 at 40 µg/mL has a MIC value of 7.5 µg/mL; it was therefore used as the control. Upon incubation at 39 °C for 30 min (pH 3) and then 2 h at pH 6, the MIC increased to 10 µg/mL (Table 6). Interestingly, Alg NPs + DRS-B2 containing menthol or lactic acid have the same antibacterial activity before treatment as the aforementioned enzymes with a MIC value of 1.25 µg/mL (Table 6). Taken together, these results indicate a protective effect of Alg NPs against the aforementioned enzymes. It should be noted that, after treatment with digestive enzymes, the MIC values of these different formulations increased, exceeding therefore those obtained without any physical or enzymatic treatment (Table 6). Related to that, MIC values increased by two-fold, which indicates that the enzymes somehow modified the DRS-B2 structure, and therefore its activity, which was also noticed on the activities of the new formulation. Interestingly, the antibacterial activity of the new formulations Alg NPs + DRS-B2 containing either menthol or lactic acid remained lower (2.5 µg/mL) upon their treatment with the digestive enzymes in comparison to formulations without these small molecules (>5 µg/mL) (Table 6). Therefore, small molecules offer protection to Alg NPs + DRS-B2 from enzymatic degradation of digestive enzymes. Indeed, upon the treatment of DRS-B2 or Alg NPs + DRS-B2 with pancreatic enzymes, the MIC values augmented from 5 to 10 µg/mL (Table 6).

To sum up, it appeared that digestive enzymes could influence the activity of DRS-B2 alone, as well as that of the new formulation composed of DRS-B2 adsorbed on Alg NPs. Nonetheless, small molecules lowered the actions of digestive enzymes, likely via a coating phenomenon [33] or another mechanism that remains to be understood.

## 4. Discussion

Here, we describe and discuss the incentives of Alg NPs and small molecules such as menthol and lactic acid in relation to the antibacterial activity of DRS-B2, a member of the DRS family. Indeed, DRS-B2 is active against *E. coli* strains, including *E. coli* 184, which harbors the famous *mcr-1* gene. Upon optimal adsorption of DRS-B2 on Alg NPs, we obtained a new formulation endowed with a remarkable anti-*E. coli* activity. Indeed, the MIC values recorded for Alg NPs + DRS-B2 are two-fold lower than those obtained with DRS-B2 alone. This potentiating effect has been previously reported [24,25,34], and here confirms the possible use of Alg NPs as enhancers of AMP activities. The adsorption of DRS-B2 on the surface of Alg NPs improved the antibacterial activity of DRS-B2. The use of Alg NPs has been discussed as a potential driver of peptides to the bacterial cell membrane. The adsorption of DRS-B2 on Alg NPs could result from electrostatic interactions between the anionic charge of the alginate and the cationic charge of the dermaseptin [9]. These interactions allow one to assemble DRS-B2 and Alg NPs within a formulation possessing a potent antibacterial activity and that could be used to deliver DRS-B2 and reach the phospholipid bilayer membrane [9]. The loading of DRS-B2 on Alg NPs generated a more active formulation that needs to be tested in vivo before its approval for therapeutic applications. The applications envisaged are objective and realizable, especially when the investments for the research of new antibiotics are in a strong decline.

Other polymers, such as maltodextrins, have been successfully used to prepare nanoparticles for pharmaceutical, agri-food, and cosmetology applications [35]. Maltodextrins are abundant in nature, and nanoparticles issued from this polymer are also deemed biocompatible and biodegradable. Their derived forms have been extensively studied in many applications, such as the delivery of anticancer proteins [36,37].

Chitosan is another polysaccharide polymer that is biocompatible and biodegradable, safe, and could be used to deliver drugs orally [38]. Chitosan is a bacteriostatic and bactericidal molecule for a wide range of Gram-positive and Gram-negative bacteria [39]. All these examples underpin the advantages of different natural polymers that could be used as vectors of bioactive molecules including AMPs, but also plant-derived molecules and phenolic compounds [40].

In spite of its potent antibacterial activity, the application of Alg NPs + DRS-B2 can be limited by the conditions encountered in the GIT environment. To check this hypothesis, the formulation Alg NPs + DRS-B2 was subjected to a set of analyses to understand whether it can still be active at low pH values and in front of digestive enzymes. Whilst the antibacterial activity of Alg NPs + DRS-B2 remained stable at different pH values, the contact with the digestive enzymes reduced their inhibitory activities. To overcome this situation, the composition of the new formulation Alg NPs + DRS-B2 was modified by the incorporation of small molecules, either menthol or lactic acid.

Menthol is a monoterpene alcohol, already used as an ingredient in some drug prescriptions [41]. Menthol has a broad spectrum of activity against Gram-positive bacteria, Gram-negative bacteria, and fungi [42]. Besides this large spectrum of activity, the use of essential oils is suitable as these complex molecules could minimize the development of bacterial resistance [43]. Furthermore, menthol has already been immobilized on diamond nanoparticles, and the resulting formulations were used to disrupt biofilm formation [44]. Menthol, like many essential oil compounds, is known to be toxic at high concentrations. However, this toxicity could be easily managed by using this molecule at low quantities, as has been done in the food and feed sectors [45,46].

Lactic acid is an organic acid classically used in the food industry as an additive, antioxidant, or acidifying agent [47]. Lactic acid is also endowed with inhibitory activities. This compound releases H^+^ ions, provoking acidification of the bacterial cytoplasm, forcing bacteria to compensate this environment by using its metabolic energy to stabilize the unbalanced proton motive force [27]. At high concentrations, lactic acid, such as the D-lactic enantiomer, can be detrimental because of its toxicity [48]. Of note, lactic acid has a long tradition of use as a food and a feed additive under E270 code number, according to regulation (EC) No 1333/2008 on food additives [49].

Consequently, we noticed that the addition of any of these small molecules enabled augmentation of the antibacterial activity of the formulation Alg NPs + DRS-B2 and stabilized its activity in the presence of digestive enzymes. These findings are important and could open promising applications, mainly to meet the One Health expectations. These new formulations, Alg NPs + DRS-B2 or Alg NPs + DRS-B2 + menthol or lactic acid as a therapeutic, fulfill environmental constraints and meet societal expectations. Related to environmental constraints, alginate is considered as a biodegradable and biocompatible polymer, already used for cell encapsulation [29,30,31]. Previous studies have reported the application of Alg NPs as a vector to ascertain drug delivery [50], as well as a biosensor molecule [51].

The safety of DRS-B2 and DRS-B2 adsorbed on Alg NPs, with or without the concomitant presence of small molecules, was ascertained. The hemolytic activity of DRS-B2 and new formulations was established on human erythrocytes and confirmed on human (HT29) and porcine (IPEC-1) models, which are usually used prior to *in vivo* assays and potential therapeutic outcomes [52,53]. Although DRS-B2 is weakly toxic and hemolytic at high concentrations, these adverse effects were neutralized when this peptide was loaded onto Alg NPs.

Of note, though many peptides endowed with biological functions, such as inhibitory activities, could be hemolytic, their combination with nanoparticles frequently allows them to discard this adverse trait [33]. Charpentier et al. [10] focused on the cytotoxicity of dermaseptin B3 and B4 on mammalian cells. These authors evaluated dermaseptin B3 and B4 abilities to inhibit thymidine incorporation into transformed human MCF-7 (breast cancer) cells [10]. The results obtained unveiled 50% cytotoxicity when DRS-B3 was used at 27.8 µg/mL (10 µM). However, Galanth et al. [9] reported that when DRS-B2 was used at 39.7 µg/mL(10 µM), there was not any toxic effect on CHO (Chinese Hamster Ovary) cells, but 50% of the CHO cells were killed by DRS-B2 at 198.5 µg/mL (50 µM) upon 24 h of incubation [9].

In terms of societal expectations, these new formulations are in good agreement with perspectives to replace antibiotics in some practices. The growing antibacterial resistance to aging antibiotics constitutes a new challenge for economically advanced societies whose pharmaceutical investments for discovering new antibiotics are becoming low. As above-stated, DRS-B2 is endowed with antibacterial activity, mainly against Gram-negative bacilli. Alginate fulfills environmental constraints, and the adsorption of DRS-B2 on the surface of Alg NPs leads to a new and potent formulation that could replace, in some practices, the use of antibiotics such as colistin. The addition of small molecules to Alg NPs + DRS-B2 formulation is fully justified. To support the advantages of Alg NPs, we have recently shown that the adsorption of colistin on such nanoparticles, provided orally, permitted an efficient protection of piglets from enterotoxigenic *E. coli* infections [34].

## 5. Conclusions

The data obtained here highlight the potentiating role of Alg NPs on DRS-B2 antibacterial activity for the first time. Following the optimal adsorption of DRS-B2 on Alg NPs, we could significantly enhance the antibacterial activity of this peptide against Gram-negative bacilli and then ascertain its stability under the conditions encountered in the GIT. When the composition of the formulation was slightly modified by adding small molecules, such as menthol or lactic acid, at non-toxic concentrations, we further improved the antibacterial activity and protected DRS-B2 from enzymatic degradation. Overall, these formulations were found to be active, safe, and stable, offering guaranties for future applications as alternatives to antibiotics.

## Figures and Tables

**Table 1:** Formulations of Alg NPs + DRS-B2 supplemented with small molecules such as menthol or lactic acid.

Formulations (on Basis of DRS-B2)	(DRS-B2) µg/mL	(Alg NPs) µg/mL	(Lactic Acid) µg/mL	(Menthol) µg/mL
F_1_	125	-	-	-
F_2_	125	500	-	-
F_3_	125	-	1	-
F_4_	125	-	-	10
F_5_	125	500	-	10
F_6_	125	500	1	-
F_7_	60	-	-	-
F_8_	60	500	-	-
F_9_	30	-	-	-
F_10_	30	500	-	-
F_11_	40	500	-	-
F_12_	40	500	-	-
F_13_	40	500	-	10
F_14_	40	500	1	-
F_15_	40	500	-	10
F_16_	40	500	1	-
F_17_	30	500	-	10
F_18_	30	500	1	-
F_19_	30	500	-	10
F_20_	30		1	-

**Table 2:** Concentrations of DRS-B2 adsorbed on Alg NPs (500 μg/mL) after dialysis.

(DRS-B2) (µg/mL) before Dialysis	Peak Area before Dialysis	Peak Are after Dialysis (Y)	(DRS-B2) (µg/mL) after Dialysis (X) *	** Percentage of Initial DRS-B2 Ad Sorbed on Alg NPs
199	4,091,385	3,954,214	198.4	40
225	4,521,891	3,998,514	200.6	40
250	5,149,931	4,031,252	202.3	40.4

X: DRS-B2 concentration (µg/mL) after dialysis; Y: Peak area after dialysis; α = 19931. * Y = 19931X; X = Y/19931; X = 3998514/19931 = 200.61 µg/mL; ** % = (200.61/500) × 100 = 40%.

**Table 3:** MIC values of formulation Alg NPs + DRS-B2 against *E. coli* strains.

		MIC (µg/mL)	
Formulations	(DRS-B2)	*E. coli* 184	*E. coli* ATCC8739
(DRS-B2) µg/mL	-	7.5	3.75
Alg NPs (500 µg/mL)	125	3.91	1.95
+	40	2.5	1.25
(DRS-B2) µg/mL	30	3.75	1.87

**Table 4:** MICs of the new formulation Alg NPs + DRS-B2 supplemented with menthol.

		MIC (µg/mL)	
Formulations	(DRS-B2)	*E. coli* 184	*E. coli* ATCC8739
Alg NPs (500 µg/mL)	125	1.95	0.97
+			
(DRS-B2) µg/mL	40	1.25	0.62
+			
Menthol (10 µg/mL)	30	1.87	0.94

**Table 5:** MICs of the formulation Alg NPs + DRS-B2 supplemented with lactic acid.

		MIC (µg/mL)	
Formulations	(DRS-B2)	*E. coli* 184	*E. coli* ATCC8739
Alg NPs (500 µg/mL)	125	1.95	0.97
+			
(DRS-B2) µg/mL	40	1.25	0.62
+			
Lactic acid (1 µg/mL)	30	1.87	0.94

**Table 6:** Effects of digestive enzymes on Alg NPs + DRS-B2 + lactic acid or Alg NPs + DRS-B2 + menthol antibacterial activities.

MICs of DRS-B2 (µg/mL)				
Enzymes Mix	Without Treatment	Treatment with pH Variation Only * (Incubation 30 min at pH 3 and Then 2 h at pH 6)	Treatment with Pepsin * (Incubation 30 min at pH 3 with Pepsin and Then 2 h at pH 6)	Treatment with Digestive Enzymes * (Incubation 30 min at pH 3 with Pepsin and Then 2h at pH 6 with Trypsin + Chymotrypsin)
DRS-B2 (40 µg/mL)	7.5	10	≥10	≥10
Alg NPs (500 µg/mL)	2.5	2.5	5	10
+				
DRS-B2 (40 µg/mL)				
Alg NPs (500 µg/mL)	1.25	1.25	2.5	2.5
+				
DRS-B2 (40 µg/mL)				
+				
Lactic acid (1 µg/mL)				
Alg NPs (500 µg/mL)	1.25	1.25	2.5	2.5
+				
DRS-B2 (40 µg/mL)				
+				
Menthol (10 µg/mL)				

* Incubation: 39 °C at 140 rpm. Enzymes were used as follows: Pepsin (15 U/mL); Trypsin (40 U/µL); Chymotrypsin (5 U/mL).

## Data Availability

Not applicable.

## Supplementary Materials

The following supporting information can be downloaded at: https://www.mdpi.com/article/10.3390/antibiotics11060787/s1, Figure S1: Variation of peak area of the different concentrations of DRS-B2 before (black rods) and after (white rods) dialysis. The concentration of Alg NPs was 500 μg/mL. Table S1: Characterisation of the size and charge (zeta potential) of Alg NPs loaded or not with DRS-B2 at pH 6.3.
