# Supplementary material for: Enhanced Antibacterial Activity of Dermaseptin through Its Immobilization on Alginate Nanoparticles—Effects of Menthol and Lactic Acid on Its Potentialization
Source: Antibiotics (Basel). 2022 Jun 9;11(6):787. doi: 10.3390/antibiotics11060787 (PMC9220408; doi:10.3390/antibiotics11060787)
Supplement: Supplementary file 1 [file antibiotics-11-00787-s001.zip › antibiotics-1756273-supplementary.pdf]

Supplementary Information

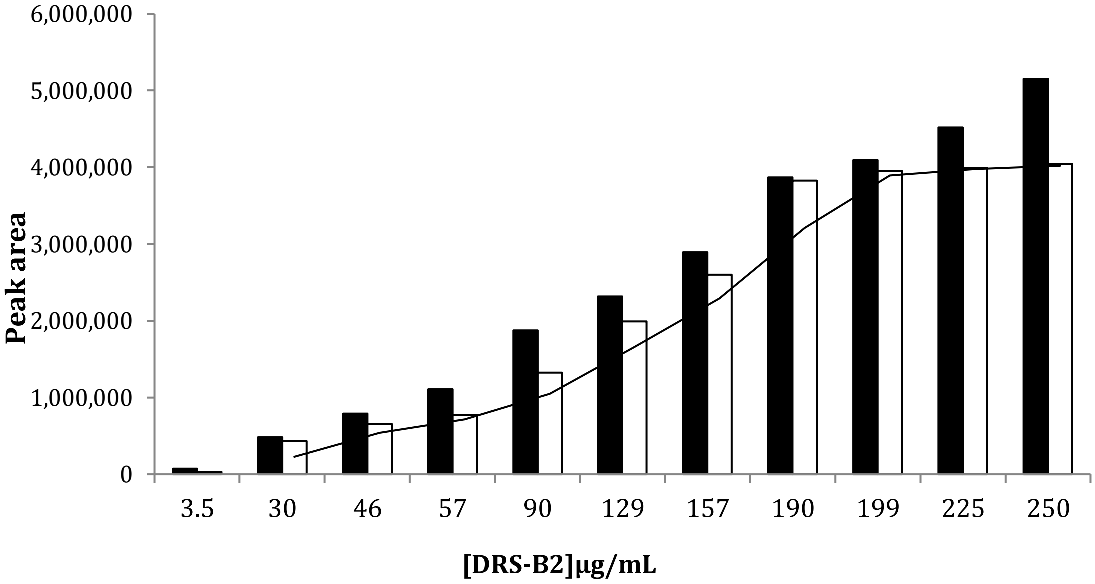

Figure S1: Variation of peak area of the different concentrations of DRS-B2 before (black rods) and after (white rods) dialysis. The concentration of Alg NPs was 500 µg/mL.

Table S1: Characterisation of the size and charge (zeta potential) of Alg NPs loaded or not with DRS-B2 at pH 6.3

Formulations	Size (nm)	Zeta Potential (ζ) (mv)
**Alg NPs (500 µg/mL)**	111.1	−22
**Alg NPs (500 µg/mL) + DRS-B2 (40 µg/mL)**	128	+0.9
**DRSB2 (40 µg/mL)**	-	+23
